# Headache‐associated photophobia is more prevalent during winter: A cross‐sectional study from a pediatric headache registry

**DOI:** 10.1111/head.70072

**Published:** 2026-03-02

**Authors:** Carlyn Patterson Gentile, Geoffrey K. Aguirre, Andrew D. Hershey, Christina L. Szperka

**Affiliations:** ^1^ Children's Hospital of Philadelphia Philadelphia Pennsylvania USA; ^2^ Perelman School of Medicine at the University of Pennsylvania Philadelphia Pennsylvania USA; ^3^ Department of Pediatrics Cincinnati Children's Hospital Medical Center and University of Cincinnati College of Medicine Cincinnati Ohio USA; ^4^ Department of Neurology Cincinnati Children's Hospital Medical Center and University of Cincinnati College of Medicine Cincinnati Ohio USA

**Keywords:** circannual variation, migraine, pediatric, photophobia

## Abstract

It remains unclear whether avoiding uncomfortable light is the best strategy to manage light sensitivity associated with migraine, or if restricting light exposure increases intolerance over time. We found that youth with migraine were more likely to report light sensitivity in the winter when light exposure tends to be lower. This finding suggests that light sensitivity is worse when light exposure is lower, but further research is needed to confirm this relationship.

**Abbreviations**
IQR: interquartile range
MESOR: Midline Estimating Statistic of Rhythm

Sensory sensitivity is commonly associated with headache. It remains unclear whether avoiding uncomfortable sensory stimuli is the best strategy to manage symptoms, or if restricting sensory exposure increases intolerance over time. Daily light exposure varies with seasons.^1^ Therefore, we aimed to determine if headache‐related photophobia is more prevalent closer to the winter solstice, when light exposure is lower, as compared to the summer solstice. We measured seasonal fluctuations in sensory sensitivity prevalence in youth presenting for headache evaluation. We hypothesized that photophobia would be more prevalent closer to the winter solstice compared to the summer solstice when light exposure is lower, whereas phonophobia would not show seasonal variation because noise exposure does not show the same seasonal fluctuation.

This single‐center cross‐sectional study measured patient‐reported photophobia and phonophobia associated with headache from questionnaires (11/2022–8/2024) collected at their first visit in pediatric general neurology and headache clinics. Age cutoff of ≥ 12 years was used given the higher prevalence of headache‐associated symptoms in older youth.^2^ Children's Hospital of Philadelphia Institutional Review Board (IRB) (Philadelphia, Pennsylvania) approved the extraction of data from the electronic health record into a research registry (IRB 14‐011369), with a waiver of consent and assent to maximize generalizability. Binary variables were reported as proportions, age was reported as median and interquartile range (IQR) due to non‐normal distribution. Statistical analyses were performed using MatLab version R2024b (MathWorks, Natick, MA, USA). No a priori statistical power calculation was conducted; the sample size was based on available data in the registry during the study period. Univariable log‐binomial regression analysis was used to determine prevalence of photophobia or phonophobia near the winter (November–January; mean daylength 9.7 h) compared to the summer (May–July; mean daylength 14.7 h) solstice.^3^ Significance was defined as *p* < 0.05, hypothesis testing was two‐tailed. To determine circannual periodicity, monthly sensory sensitivity prevalence was subjected to cosinor logistic regression. No adjustments for missing data were made because missingness was low (4.9%).

A total of 2040 questionnaires (72.5% female, median age 15 years [IQR 13, 16]) were included. Photophobia prevalence was significantly higher in the winter compared to summer (Figure 1A) (prevalence ratio 1.10 [95% confidence interval (CI) 1.02, 1.19], *p* = 0.020). No seasonal difference was observed for phonophobia (prevalence ratio 1.02 [95% CI 0.92, 1.14], *p* = 0.690). A significant circannual variation in photophobia prevalence was observed (cosine, $\beta_{1}$ *p* = 0.004; sine, $\beta_{2}$ *p* = 0.192) with a midpoint of 0.70, an amplitude of 0.05, and a peak at 0.9 months [95% CI 11.7, 2.0] (Figure 1B). There was not significant circannual periodicity for phonophobia (cosine, $\beta_{1}$ *p* = 0.620; sine, $\beta_{2}$ *p* = 0.855).

**FIGURE 1:**
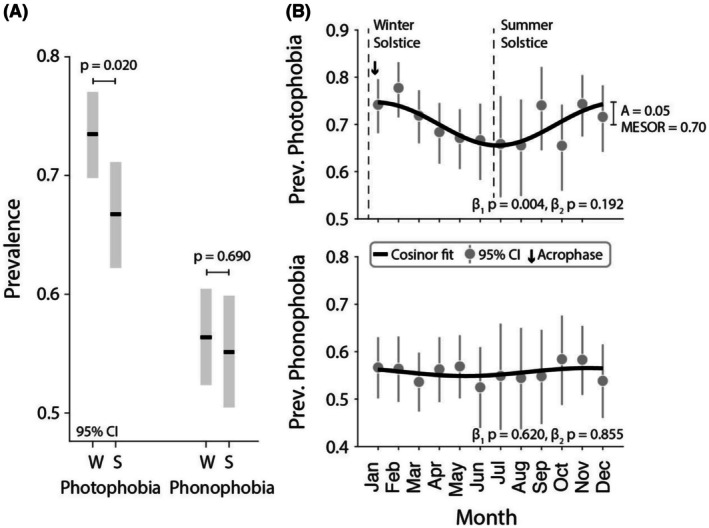
Prevalence of photophobia and phonophobia by season. (A) The prevalence of photophobia and phonophobia over the 3 months surrounding the winter solstice and summer solstice is shown with 95% confidence intervals. (B) Monthly prevalence of photophobia (top) and phonophobia (bottom) fitted to a cosinor logistic regression function. A, amplitude; Apr, April; Aug, August; Dec, December; Feb, February; Jan, January; Jul, July; Jun, June; Mar, March; MESOR, midline estimating statistic of rhythm; Nov, November; Oct, October; Prev., prevalence; S, summer solstice; Sep, September; W, winter solstice.

We found a circannual periodicity to the prevalence of photophobia, which was higher in the winter. No seasonal variation was observed in phonophobia prevalence, strengthening the assertion that the seasonal increase in photophobia is associated with decreased daily light exposure. These findings are opposite from Bekkelund and colleagues, who found interictal photophobia and migraine frequency were worse in adults in the subarctic bright summer months,^4^ where seasonal daylength fluctuations are more extreme. One explanation is prolonged light exposure unique to the polar summer suppresses melatonin leading to circadian dysfunction.^4^ Another factor to consider is the impact of school. We did see an increase in photophobia in September (school year start). Possible explanations include a shift from outdoor to indoor lighting environments, increased screen time, or changes in circadian habits when transitioning from a summer to school schedule. However, photophobia prevalence started decreasing in March during the school year and steadily declined reaching a nadir at the start of summer break, indicating school cannot fully account for seasonal variations in photophobia prevalence. Study limitations include patient report, which is subject to recall bias, and measurement at a single time point, which did not allow us to determine within‐participant seasonal fluctuations in light exposure. Further study is needed to determine generalizability to different geographic locations and ages. Our findings support the proposal that lower daily light exposure increases light intolerance. Seasonal differences in light exposure are likely multifactorial (e.g., time spent outside, daylength), and longitudinal studies with objective light exposure measurements are needed to unpack the seasonal variation of photophobia prevalence.

## AUTHOR CONTRIBUTIONS

**Carlyn Patterson Gentile:** Conceptualization; writing – original draft; visualization; software; formal analysis; methodology; data curation. **Geoffrey K. Aguirre:** Writing – review and editing; methodology. **Andrew D. Hershey:** Resources; supervision; writing – review and editing. **Christina L. Szperka:** Resources; supervision; writing – review and editing.

## FUNDING INFORMATION

This work was supported by the National Institutes of Health National Institute of Neurological Disorders and Stroke (K23NS124986 to C.P.G).

## CONFLICT OF INTEREST STATEMENT

**Carlyn Patterson Gentile** is currently or recently funded by the National Institutes of Health/National Institute of Neurological Disorders and Stroke (K23 NS124986) and the Children's Hospital of Philadelphia Foerderer Institutional grant. **Andrew D. Hershey** or his institution have received compensation for serving as a consultant for AbbVie, Amgen, Biohaven, Eli Lilly, Lundbeck, Supernus, Teva, Theranica, and Upsher‐Smith. His institution has also received research support from Amgen, Biohaven, Eli Lilly, Theranica, Upsher‐Smith, and the National Institutes of Health (NIH) National Institute of Neurological Disorders and Stroke/National Institute of Child Health and Human Development. **Geoffrey K. Aguirre** receives funding/grant support from the National Institute of Neurological Disorders and Stroke, the National Eye Institute, and the Binational Science Foundation. **Christina L. Szperka** has received research/grant support from the Patient‐Centered Outcomes Research Institute. Dr. Szperka or her institution have received compensation for her consulting work for Eli Lilly, Teva Pharmaceutical Industries, Upsher‐Smith Laboratories, Abbvie, and Lundbeck.

## References

[1] Thorne HC , Jones KH , Peters SP , Archer SN , Dijk DJ . Daily and seasonal variation in the spectral composition of light exposure in humans. Chronobiol Int. 2009;26:854‐866. 19637047 10.1080/07420520903044315

[2] Patterson Gentile C , Szperka CL , Hershey AD . Cluster analysis of migraine‐associated symptoms (CAMS) in youth: a retrospective cross‐sectional multicenter study. Headache. 2024;64:1230‐1243. 39463035 10.1111/head.14859 PMC11560594

[3] United States Naval Observatory . Duration of Daylight/Darkness Table. 2025. Accessed June 30, 2025. https://aa.usno.navy.mil/calculated/durdaydark?year=2025&task=0&lat=39.9526&lon=‐75.1652&label=Philadelphia%2C+PA&tz=0.00&tz_sign=‐1&submit=Get+Data

[4] Bekkelund SI , Müller KI , Wilhelmsen A , Alstadhaug KB . Photophobia and seasonal variation of migraine in a subarctic population. Headache. 2017;57:1206‐1216. 28631303 10.1111/head.13131

